# A profile of adult patients with major burns admitted to a Level 1 Trauma Centre and their functional outcomes at discharge: A retrospective review

**DOI:** 10.4102/sajp.v78i1.1543

**Published:** 2022-01-11

**Authors:** Irene K. Angelou, Heleen van Aswegen, Moira Wilson, Regina Grobler

**Affiliations:** 1 Department of Physiotherapy, Faculty of Health Sciences, University of the Witwatersrand, Johannesburg, South Africa; 2 Department of Physiotherapy, Netcare Milpark Hospital, Johannesburg, South Africa; 3 Trauma Division, Netcare Hospital Group, Johannesburg, South Africa

**Keywords:** burn injury, physical function, length of stay, injury severity, range of motion

## Abstract

**Background:**

Patients with major burns suffer with pain, which impacts their physical function during hospitalisation.

**Objectives:**

To describe the demographics, burn characteristics, clinical course, physical function, complications developed after major burns and to establish predictors of non-independent physical function at hospital discharge.

**Method:**

Records of all consecutive adult burn admissions to a Level 1 Trauma Centre between 2015 and 2017 were screened retrospectively against our study criteria, using the Trauma Bank Data Registry. Anonymised data from included records were captured on specifically designed data extraction forms. Descriptive statistics were used to summarise findings. A regression analysis was undertaken to establish predictors of non-independent function at discharge.

**Results:**

Males represented 87.7% (*n* = 64) of included records (*n* = 73). Median age was 38 (interquartile range [IQR]: 22). Thermal burns were most reported (*n* = 47, 64.4%), followed by median total body surface area (TBSA) 31% and head and arms were most affected (60.3% and 71.2%). Injury severity was high with median intensive care unit (ICU) length of stay (LOS) of 17 (IQR: 34) and hospital LOS 44 (IQR: 31) days. Wound debridement was mostly performed (*n* = 27, 36.9%) with limb oedema as a common complication (*n* = 15, 21.7%). Muscle strength and functional performance improved throughout LOS. None of the variables identified were predictors of non-independent function at hospital discharge.

**Conclusion:**

Adults with major burns were predominantly male, in mid-life and sustained thermal injury with a high injury severity. Decreased range of motion (ROM) of affected areas, ‘fair’ muscle strength and independent function were recorded for most patients at hospital discharge.

**Clinical implications:**

These findings contribute to the limited body of evidence on the profile, clinical course and outcomes of South African adult burn patients.

## Introduction

Globally, burn injuries cause approximately 180 000 deaths per year and are the leading cause of morbidity in cases that are not fatal (Forjuoh 2006; Smolle et al. 2017; World Health Organization [WHO] 2019). In middle- to low-income countries, one of the main causes of disability-adjusted life years lost are burn injuries (WHO 2019), specifically burns caused by fire (Den Hollander et al. 2014). Approximately one million people in Africa sustain burn injuries every year (Rode, Berg & Rogers 2011). Men have a higher probability of sustaining burn injuries, but women with burn injuries present with a higher mortality rate than men (Smolle et al. 2017; WHO 2019). Major burn injury is defined as injury to 20% or more of the total body surface area (TBSA) (excluding superficial burns) and with more than 5% being full thickness injuries (Borke, Zieve & Ogilvie 2016; Gauglitz & Williams 2016; Hettiaratchy & Papini 2004). This is compounded by inhalation injuries or major trauma, high voltage electrical burns, chemical burns and lastly, second-degree burns over the major joints, buttocks, feet, face or hands (Borke et al. 2016; Gauglitz & Williams 2016; Hettiaratchy & Papini 2004). Inhalation injury is mostly confined to the upper airway and raises the risk of mortality eightfold compared with patients with burn injuries without inhalation injury (Esselman 2007; Hettiaratchy & Dziewulski 2004).

Although there is a declining trend in burn injuries in South Africa, these injuries remain distressing because they cause varying degrees of morbidity, emotional upheaval and impaired quality of life (Smolle et al. 2017). Burn injuries predominantly occur in the workplace, home or outdoors. Many South Africans use kerosene as a home energy source, which is directly related to a higher incidence of household fires (Maritz et al. 2012; Rode et al. 2011). Burn injuries may be unintentional, self-inflicted or sustained during assault or abuse (Forjuoh 2006). Injury sustained because of thermal burns often requires hospital admission because of its higher level of severity (Den Hollander et al. 2014; Rode et al. 2011, 2013).

Systemic changes occur in patients with burn injuries of 30% TBSA or more and allow for complications to develop (Corner et al. 2015; Hettiaratchy & Dziewulski 2004; Orgill 2009). Firstly, immunosuppression develops, which increases a patient’s vulnerability to sepsis and multiple organ failure in response to systemic inflammatory response syndrome (Nielson et al. 2017; Strudwick & Cowin 2018). Secondly, malnutrition develops on account of impaired endocrinal function, affects wound healing and precipitates infection (Hettiaratchy & Dziewulski 2004; Kasten, Makley & Kagan 2011; Orgill 2009). In a South African context, 46% of patients with burn injury present with malnutrition at hospital admission, and after 7 days in hospital, an additional 16% present with malnutrition (Kingu et al. 2011). Thirdly, muscle catabolism and deconditioning develop because of the hypermetabolic state associated with major burn injury and duration of immobility because of pain and discomfort experienced. This predisposes patients to varying degrees of disability, which might last from 6 months to several years following injury (Corner et al. 2015; De Lateur et al. 2007; Greenhalgh 2017; Hanekom et al. 2015; Hettiaratchy & Dziewulski 2004; Nielson et al. 2017). Neuromusculoskeletal complications known to develop following major burn injury include loss of lean body mass, decreased functional ability, decreased active joint range of motion (ROM), joint deformities, heterotopic ossification, hypertrophic scarring and contractures (Ault, Plaza & Paratz 2017; Esselman 2007; Hanekom et al. 2015; Leblebici et al. 2006).

Management of patients with or without major burn injury includes analgesia, antibiotic and antifungal therapy, intubation and mechanical ventilation and sedation as required, wound care, splinting of affected areas, positioning, exercise therapy and rehabilitation (Hanekom et al. 2015). Surgery is indicated for deep partial and full thickness burns, contaminated burns, circumferential burns and may require excision of necrotic tissue to reduce the risk of mortality (Alharbi et al. 2012; Giaquinto-Cilliers et al. 2014). Patients with burn injuries have prolonged hospital length of stay (LOS) and LOS equates to 2 days per percentage of TBSA for burns more than 50% (Den Hollander et al. 2014).

Existing evidence of burn management and rehabilitation in South Africa reports on the incidence of acute malnutrition and predictors of mortality in adult and paediatric burn patients (Kingu et al. 2011); epidemiology and mortality outcome of adult and paediatric patients (Den Hollander et al. 2014); burn mortality across their lifespan (Van Niekerk, Laubscher & Laflamme 2009); challenges experienced by surgeons in the management of patients with burns (Rode et al. 2011, 2013); a profile of paediatric patients with burns (Parbhoo, Louw & Grimmer-Somers 2010) and the use of gaming technology as an adjunct to physiotherapy management of paediatric patients with burns (Lozano & Potterton 2018).

Published studies on the profiles and management of burn populations in South Africa have been conducted in public sector healthcare settings in the Eastern Cape (Kingu et al. 2011), Western Cape (Parbhoo et al. 2010; Van Niekerk et al. 2009), KwaZulu-Natal (Den Hollander et al. 2014) and Gauteng (Lozano & Potterton 2018) provinces. The majority of these studies included paediatric patients or were performed exclusively on paediatric patients. No information is available on the profile of adult patients with major burn injuries admitted to a private healthcare Level 1 Trauma Centre or their clinical course and functional outcomes during hospitalisation. Thus, the aim of our study was to present a profile of adult patients with major burn injuries admitted to a Level 1 Trauma Centre and their functional outcomes at hospital discharge. This profile includes the demographics and burn characteristics, clinical course, physical function and complications developed by adult patients with major burn injuries. We also aimed to identify predictors of non-independent function at hospital discharge.

## Method

A retrospective record review of adults who had sustained major burn injuries and were admitted to a Level 1 Trauma Centre, over a 36-month period (January 2015–December 2017) was conducted. The Trauma Bank Data Registry was used to identify all consecutive patients admitted to the Burns intensive care unit (ICU) during this period. Screening of records was performed by the trauma research nurse against the study inclusion criteria: patients with burns of 20% TBSA or more (excluding superficial burns) with or without an inhalation injury (Gauglitz & Williams 2016). The exclusion criteria included patients with complex lower limb injuries (e.g. those who had an amputation because of the extent of burn injury or complex fractures to the pelvis, femur, tibia and fibula that delayed mobilisation), spinal cord injury, cognitive disorders (e.g. dementia, traumatic brain injury) and patients who had died in hospital. Patients with records that fitted our study inclusion criteria were contacted telephonically to explain the purpose of the study to them and to obtain their permission to include their anonymised information from the Trauma Bank Data Registry and their physiotherapy records in our study.

On receipt of permission, information retrieved from the Trauma Bank Data Registry for the study included: TBSA (determined using the Rule of Nines), Injury Severity Score (ISS) on admission, Baux or revised Baux score (prediction of mortality), age, gender, ethnicity, type of burn, surgical procedures performed during hospitalisation, ICU LOS and hospital LOS. The following information was retrieved from the physiotherapy records of the study participants who provided consent: functional ability (Functional Status Score for the ICU, [FSS-ICU]), intubation and mechanical ventilation, sedation, areas affected by the burn injury, joint ROM, muscle strength, mobilisation, stair climbing and recorded complications developed (as this information was not captured in the Trauma Bank Data Registry). All information retrieved was anonymised before it was captured on the study-specific data extraction forms.

Time points for data collection were patient admission to the Burns ICU, patient transfer to the general ward and at discharge from the hospital. Verification of the data-capturing process was performed by one of the authors on a portion of the data to ensure accuracy of data capturing.

## Statistical analysis

Data were captured on an Excel spreadsheet. Incomplete data were managed by carrying through the last-observation-forward method. Data were imported into the IBM Statistical Package for the Social Sciences (SPSS) software programme (version 25). Descriptive statistics were used to summarise the information obtained. Normality of the distribution of data was assessed using the Shapiro–Wilk test. Continuous variables were summarised as means and standard deviations (SD) for normally distributed data or as medians and interquartile ranges (IQR). Categorical data were summarised using numbers and percentages. Predictors of non-independent function at hospital discharge were calculated using binary logistic regression analysis. A priori selected variables included gender, ISS (≤ 16 or > 16), number of theatre visits (≤ 6 or > 6), and number of complications developed (≤ 2 or > 2) as dichotomous variables and ICU LOS and hospital LOS as continuous variables. Testing was performed at a level of significance of 5% (*p* ≤ 0.05) and 95% confidence intervals.

### Ethical considerations

Ethical clearance was obtained from the University of Witwatersrand Research Ethics (Medical) Committee (clearance number: M171007). Permissions were received from the Trauma Society of South Africa (TSSA) accredited Level 1 Trauma Centre and Burns Unit at a private hospital in Johannesburg, South Africa, the hospital group research committee, hospital management, trauma programme manager and physiotherapy practice owner (that services the Burns intensive care unit and wards at the Level 1 Trauma Centre) gave permission to conduct our study.

## Results

**FIGURE 1:**
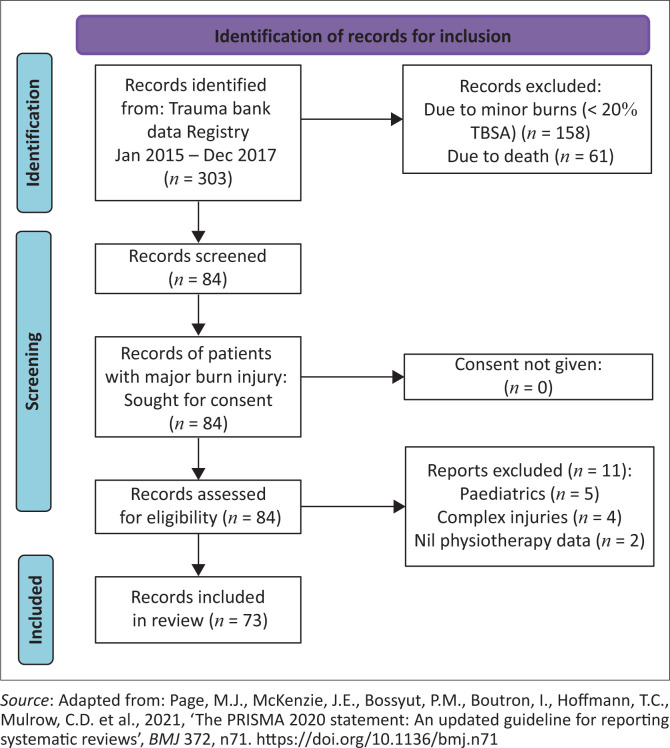
Diagram of records screened and included in our review.

A total of 303 patients were admitted to the Burns ICU during the study period. The records of 73 patients fitted the inclusion criteria (Figure 1).

### Demographics and burn characteristics

A profile of the cohort’s admission demographics and burn characteristics is summarised in Tables 1 and 2.

**TABLE 1:** Demographic information of patients admitted to the Burns intensive care unit with major burn injury during the period 2015–2017 (*n* = 73).

Variable	Results	
	*n*	%
**Age (years)**		
Median (IQR)	38.0	22.0
Minimum	19.0	-
Maximum	88.0	-
**Ethnicity**		
Black	42.0	57.5
White	31.0	42.5
**Gender**		
Female	9.0	12.3
Male	64.0	87.7
**Injury Severity Score**		
Median (IQR)	16.0	16.0
Minimum	1.0	-
Maximum	32.0	-
**Baux score (mean, SD)**	**78.5**	**22.2**
**Revised Baux score (mean, SD)**	**93.0**	**19.3**

IQR, interquartile range; *n*, number; %, percentage; SD, standard deviation.

**TABLE 2:** Burn characteristics of patients admitted with major burn injury (*n* = 73).

Variable	Results	
	*n*	%
**Type of burn injury sustained**		
Chemical	8	10.9
Electrical	18	24.7
Thermal	47	64.4
**Body areas affected**		
Arms	52	71.2
Head	44	60.3
Legs	36	49.3
Hand and wrist	31	42.5
Neck	26	35.6
Chest	25	34.3
Back	23	31.5
Elbows	19	26
Abdomen	15	20.5
Hips	15	20.5
Shoulders	14	19.2
Ankle and foot	11	15.1
Flank	8	11.0
Knees	8	11.0
**Inhalation injury**	17	23.3
**TBSA**		
Median (IQR)	31	20.0
Minimum	20	-
Maximum	77	-

IQR, interquartile range; *n*, number; %, percentage; TBSA, total body surface area.

An Injury Severity Score (ISS) of more than 15 is associated with a 10% increased risk of mortality (Karadsheh & Taylor 2018). Similarly, a revised Baux score of more than 75 is associated with a 50% mortality risk (Smith, Allorto & Clarke 2016). The risk of mortality was therefore increased in this cohort.

Thermal burns were the most common type of burns sustained and injury to the arms and head were most recorded.

### Course during hospital stay

The records of all 73 participants indicated that they were directly admitted into the Burns ICU and only transferred to the general ward once their condition had stabilised. A summary of the cohort’s clinical course during their hospital stay is provided in Table 3.

**TABLE 3:** Clinical course of the cohort during their hospital stay (*n* = 73).

Variable	Results	
	*n*	%
**Sedation**	33	45.2
**Intubation and MV**	32	43.8
**Re-intubation**	3	4.1
**ICU LOS (days)**		
Median (IQR)	17	34
Minimum	0	-
Maximum	81	-
**Theatre visits**		
Median (IQR)	6	9
Minimum	0	-
Maximum	29	-
**Complications**		
Median (IQR)	2	4
Minimum	0	-
Maximum	21	-
**Hospital LOS (days)**		
Median (IQR)	44	31
Minimum	7	-
Maximum	243	-

ICU, intensive care unit; IQR, interquartile range; LOS, length of stay; MV, mechanical ventilation; *n*, number; %, percentage.

The two most performed surgical procedures for this patient cohort were wound debridement (*n* = 27, 37%) and skin grafts (*n* = 22, 30.1%). The most common complication reported, in the physiotherapy records, for this cohort was oedema formation (*n* = 15; 21.7%). Other reported complications included pyrexia (*n* = 13, 18.8%), low haemoglobin (*n* = 11, 15.9%), diarrhoea (*n* = 10, 14.5%), blisters and infection (*n* = 8, 11.6%).

The records of all patients included in our review indicated that they received standard physiotherapy management during their hospital stay. A total of 15 patients (21.1%) received splinting as part of their burn management. Small changes in joint ROM of affected areas were recorded at the time of transfer from ICU to the general ward and prior to hospital discharge (Table 4).

**TABLE 4:** Joint range of motion changes recorded at intensive care unit discharge and prior to hospital discharge for affected areas (*n* = 73).

Affected areas	ICU discharge				Hospital discharge			
	Reduced ROM		Normal ROM		Reduced ROM		Normal ROM	
	*N*	%	*N*	%	*N*	%	*N*	%
Neck (*n* = 26)	22	84.6	4	16.4	21	80.7	5	20.5
Shoulders (*n* = 14)	10	71.4	4	28.8	9	64.3	5	35.7
Elbows (*n* = 19)	15	78.9	4	23.3	13	68.4	6	31.5
Hands and wrists (*n* = 31)	25	80.6	6	19.2	23	74.2	8	25.8
Hips (*n* = 15)	13	86.7	2	15.1	12	80.0	3	20.0
Knees (*n* = 8)	6	75.0	2	25.0	6	75.0	2	25.0
Feet and ankles (*n* = 11)	9	81.8	2	18.2	9	81.8	2	18.2

ICU, intensive care unit; ROM, range of motion; *N*, number; %, percentage.

Most patients presented with ‘fair’ muscle strength, as indicated in their physiotherapy records, at the time of transfer from ICU to the general ward and prior to discharge. Two patients had bilateral shortened Achilles’ tendons and two patients had bilateral reduced axillary spacing at hospital discharge. The median FSS-ICU scores at transfer from ICU and prior to hospital discharge were 35/35 (*n* = 38, IQR: 0, minimum: 12, maximum: 25) and 35/35 (*n* = 47, IQR: 0, minimum: 22, maximum: 35), respectively. Prior to hospital discharge, 39 (53.4%) patients were recorded to be independently mobile and 21 (28.8%) were independent with stair climbing.

### Predictors of non-independent function at hospital discharge

None of the variables identified were predictors of non-independent function (FSS-ICU < 35) at hospital discharge as the *p*-values were not significant (Table 5).

**TABLE 5:** Binary logistic regression model assessing variables and their effect on non-independent physical function (FSS-ICU score < 35) at hospital discharge.

Variable	SE	95% CI	Exp(B)	*p*
Gender	1.250	0.211–28.390	2.448	0.474
ISS	0.973	0.066–2.982	0.443	0.403
ICU LOS	0.024	0.957–1.054	1.005	0.853
Number of complications	0.974	0.075–3.395	0.503	0.481
Number of theatre visits	1.178	0.569–57.535	5.721	0.139
Hospital LOS	0.014	0.963–1.018	0.990	0.491

SE, standard error; CI, confidence interval; exp(B), exponentiation of the coefficients; ISS, Injury Severity Score; FSS-ICU, Functional Status Score for the ICU; ICU, intensive care unit; LOS, length of stay.

## Discussion

Our retrospective record review provides a profile of adult patients with major burn injury admitted to a Burns ICU of a Trauma Society of South Africa (TSSA) accredited Level 1 Trauma Centre in a private healthcare setting. A summary of their clinical course and specifically the report on changes in their physical function during hospitalisation contributes to the limited body of evidence available for adult survivors of major burns in a South African context. The majority of this cohort was male, of middle age and more than half of the cohort was of black ethnicity. Thermal injury and injury to the head, arms and legs were most reported. Over 40% of patients required intubation and mechanical ventilation and sedation therapy and high ISSs increased their risk of mortality. Wound debridement was frequently performed and some patients were managed with splinting. Most patients presented with decreased joint ROM of affected areas and ‘fair’ muscle strength at hospital discharge. Only a small number of patients had contracture formation of the Achilles’ tendons and axillary spaces. Independent functional ability was recorded for most and almost a third of patients climbed stairs independently at hospital discharge.

The gender ratio in our cohort favoured males, which contrasts with an existing South African study that reported a 1:1 ratio for adult males and females who sustained burn injuries and were admitted to a tertiary hospital in KwaZulu-Natal (Den Hollander et al. 2014). The KwaZulu-Natal study included adult and paediatric patients with burn injury and was not specific to major burns (Den Hollander et al. 2014). Age ranges compare well with those reported for adults in the KwaZulu-Natal cohort (Den Hollander et al. 2014). The high incidence of thermal burns observed is similar to that reported for adult patients (68%) in the KwaZulu-Natal cohort; however, a higher incidence of inhalation injury was observed compared with the 14.3% reported in the KwaZulu-Natal study for their adult patients. The median percentage of TBSA injured is higher than that reported for adults in the KwaZulu-Natal cohort (18.5%) who had a shorter hospital LOS (22 days).

The areas that were most affected by burn injury were the head and upper and lower limbs. Not many studies report on the location of a burn, but it has been observed that the burning of victims’ clothing principally affects the lower limbs (Forjuoh 2006). In South Africa, fires are used for cooking, as a source of heat and lighting, and for social gatherings, and in most cases, injury through fire is unintentional. As people, in making and working with fire, use their hands, in the event of an accident, it is likely that the head and upper limbs are the most common areas to be injured. Our cohort comprised mostly of males who were in mid-life and thus it is reasonable to assume that their injuries sustained are associated with injury on duty or injury in the home environment (Forjuoh 2006; Hanekom et al. 2015; WHO 2019).

Wound management was the primary reason for surgery performed and most notably wound debridement. Our record review included only patients with major burns, which could explain the high rate of wound debridement surgery performed and the longer hospital LOS. Oedema formation (generalised or local) was the most recorded complication. Oedema formation is a known tissue response to burn injury and surgical intervention and is therefore an expected finding (Edgar et al. 2011; Infanger et al. 2004).

Small or no improvements in joint ROM were recorded for affected areas at patients’ transfer from ICU to the general ward and prior to hospital discharge despite physiotherapy management being given to all patients during hospitalisation. Early physiotherapy rehabilitation of patients admitted with burn injury to prevent complications associated with active joint ROM is supported by other studies (Hanekom et al. 2015; Schneider et al. 2006). It is possible that lack of patient compliance with physiotherapy management or the repeated need for joint immobilisation after multiple surgeries contributed to the decreased ROM recorded at these time points. Most patients had recorded muscle strength of ‘fair’ at hospital discharge. It is reasonable to conclude that they responded clinically to the rehabilitation received for regaining their muscle strength. Ahmed, Abdel-Aziem and Ebid (2011) observed that a structured exercise programme, specifically isokinetic training, improves eccentric and concentric muscle strength in patients with burn injuries.

At hospital discharge, more than half of our cohort were functionally independent and some were able to climb stairs independently. Patients with burn injury in the United Kingdom had low levels of physical function on admission to a Burns ICU (Corner et al. 2015). Their functional abilities improved as they were discharged from ICU and improved further prior to hospital discharge; however, few had returned to their pre-morbid level of physical function at hospital discharge (Corner et al. 2015). As no information on pre-morbid functional ability was available in the records reviewed, it is not possible to draw further conclusions about the level of independent function that was achieved at hospital discharge. Adults recovering from burn injury present with decreased peripheral muscle strength and limitations in exercise endurance at hospital discharge when compared with age- and gender-matched healthy subjects (Ozkal et al. 2020). A recently published systematic review on the predictors of health-related quality of life (QOL) after burn injury highlights the significant psychological impact that trauma has on patient recovery (Spronk et al. 2018). Therefore, patients recovering from major burn injury should be encouraged to continue attending rehabilitation services after hospital discharge to address the physical and psychological problems that might develop in the months following discharge (Ozkal et al. 2020; Spronk et al. 2018).

Limitations to our review include its retrospective nature, few records of female patients and the fact that it was conducted at a single centre. Interpretation of findings is therefore limited to this setting only. Future research should be of a prospective nature and intervention-based to investigate the effect of identified management strategies used during hospitalisation on outcomes of South African adults recovering from major burn injury to further inform clinical practice.

## Conclusion

Adults admitted with major burns to a Burn ICU of a private trauma centre are predominantly male, in mid-life and sustained thermal injury. They have a high injury severity, and some may require intubation, mechanical ventilation and sedation. Wound debridement is frequently performed, most present with oedema and the hospital stay is prolonged. Decreased ROM of affected areas, ‘fair’ muscle strength and independent function is recorded for most patients at hospital discharge.
